# Posture Evaluation of Firefighters During Simulated Fire Suppression Tasks

**DOI:** 10.1177/21650799231214275

**Published:** 2023-11-24

**Authors:** Tara Kajaks, Christina Ziebart, Vickie Galea, Brenda Vrkljan, Joy C. MacDermid

**Affiliations:** 1 Department of Kinesiology, McMaster University; 2 School of Physical Therapy, Western University; 3 School of Rehabilitation Science, McMaster University; 4 McFarlane Hand and Upper Limb Centre, St. Joseph’s Health Care London

**Keywords:** firefighters, ergonomics, posture analysis, OWAS

## Abstract

**Background:**

Posture mechanics during fire suppression tasks are associated with musculoskeletal injuries in firefighters.

**Methods:**

This study uses the Ovako Working Posture Analyzing System (OWAS) ergonomics tool to describe and evaluate the postures of 48 firefighters during 3 simulated tasks: (a) hose drag, (b) hose pull, and (c) high-rise pack lift. Ergonomics intervention prioritizations based on the OWAS action classification (AC) scores were identified using Wilcoxon signed-rank tests. Chi-square analyses identified associations between firefighter characteristics and OWAS AC scores.

**Findings:**

The initial hose pick-up phase of each task was identified as a high priority for ergonomics intervention (OWAS AC = 4) in 45.8%, 54.2%, and 45.8% of cases for Tasks 1, 2, and 3, respectively. Lower BMI was associated with higher AC scores for the initial hose pick-up during Task 3 (likelihood ratio = 9.20, *p* value = .01).

**Conclusion:**

The results inform ergonomics priorities for firefighter training based on the tasks analyzed. *Application to Practice*: This study evaluates the posture mechanics of three commonly performed firefighting tasks. The results help inform an ergonomics training intervention focused on posture mechanics during occupational activities for firefighters.

## Background

Firefighting carries many risks related to both environmental dangers and physical job demands. In Canada, firefighting is a leading occupation for lost-time claims (Workplace Safety & Insurance Board [WSIB], 2016), with sprains and strains to the back, knee, and shoulder regions accounting for many of these musculoskeletal disorders (MSDs) (Dworsky et al., 2021; Frost et al., 2016; Kajaks & MacDermid, 2015; Nazari et al., 2020; Negm et al., 2017; Tucker & Keefe, 2020; Widman et al., 2018). A recent analysis of data from a Canadian urban fire association showed that MSDs occur at the station (30.8%) or training site (27.7%), rather than during fire (18.2%) or non-fire (18.2%) emergency calls (Frost et al., 2016). Motion patterns causing these MSDs included: (a) bending, lifting, and/or squatting, (b) slipping, tripping, and/or falling, (c) lunging and/or stepping, and (d) exercise and/or training activities. These findings are corroborated in a 2019 critical review of the literature (Orr et al., 2019). Of the estimated 66,200 injuries that occurred among U.S. firefighters between 2012 and 2014, overexertion and strain were the most prevalent (27.4%), with most of these MSDs affecting firefighter upper (21.2%) and lower (20.6%) extremities (U.S. Department of Homeland Security, 2011). In an updated article specifically focusing on firefighters in California, lower extremity and trunk injuries continue to be the most common MSD, with the prevalence of injuries rising over time. In 2005, the injury rate was 200 per every 1,000 firefighters, and in 2017, it was 250 per 1,000 (Dworsky et al., 2021). Fire suppression and suppression support activities accounted for 52.0% and 23.6% of these MSDs, respectively. This high firefighter MSD and pain prevalence indicates that further MSD prevention strategies are needed.

MSDs sustained during typical firefighting tasks can be reduced through re-design of the activity itself (Lavender et al., 2015) and/or improving personal protective equipment (Park et al., 2015; Park & Hahn, 2014; Turner et al., 2010; Wang et al., 2021). However, firefighters also require the necessary training and physical attributes to perform tasks safely (Walker et al., 2014). In recent years, firefighter legislation has required that candidates pass rigorous pre-employment entrance exams, such as the Canadian Physical Assessment Test (CPAT), that help ensure that they meet job fitness standards at the time of hire. For currently employed firefighters, health and fitness maintenance guidelines (Michaelides et al., 2008, 2011; Pawlak et al., 2015) are available through many North American firefighter associations, and worldwide(Ras et al., 2022, 2023), to help promote healthy lifestyles and workplace safety.

Poor health and fitness can result in sub-standard job performance, which is a serious health and safety concern for both the firefighters and the communities they serve. Poplin et al. (2016) showed that firefighters who had a low comprehensive fitness score, which was based on cardiovascular fitness, endurance, flexibility, muscular strength, and body composition, were at greater risk of injury compared with their more fit counterparts (Poplin et al., 2016). Conversely, a study of firefighter fitness in Hamilton, Ontario, Canada, where this study was conducted, found that firefighters had similar fitness to age-matched norms (Nazari et al., 2019). Near misses (Neitzel et al., 2016) and body mass index (BMI) has also been identified as a modifiable risk factor for MSD-related injuries (Jahnke et al., 2013; Neitzel et al., 2016); however, BMI might not be the most appropriate metric in this population and should be interpreted with caution.

Age may also be an important consideration in the evaluation of firefighter MSD risk factors. Poplin et al. (2016) found that younger firefighters who were less fit had a greater MSD risk than older colleagues, although this relationship was inferred to result more from task requirements and hazard profile associated with this occupation than physiological reasons (Poplin et al., 2016). This was confirmed by female firefighters, who told us that, in recognition of their lower strength, they had to be more cognizant of their task strategies (Nazari et al., 2019). Lett and McGill (2006) demonstrated that years of experience as a firefighter contributed to less spinal compression and shearing forces during pushing and pulling tasks, which suggests with experience firefighters alter their task strategies (Lett & McGill, 2006). Conversely, Katsavouni et al. (2014) found that those with more than 5 years of work experience were at a greater risk of low back pain compared with less experienced workers (Katsavouni et al., 2014), which is corroborated by Widman et al. (2018) who found that 70% of all firefighter injuries occur in the first 15 years of experience (Widman et al., 2018). As well, a survey of 249 firefighters in Texas and California found that firefighters who reported occupational injuries were more likely to be older. They attribute this finding to occupational stressors (Phelps et al., 2018). Furthermore, they found that injured workers were more likely to have less job rewards, overcommitment, less esteem, and fewer promotional opportunities (Phelps et al., 2018). Thus, altered task strategies over time may be related to experiential changes in emphasis on prevention or in response to cumulative injury.

While it is important to understand the anthropometric, demographic, and health-related risk factors for MSDs, a better understanding of firefighter posture strategies specific to fire suppression tasks is needed before MSD-reduction ergonomics training modules can be developed. The purpose of this study was to describe and evaluate firefighter posture strategies, and associated risks, using video observation in combination with the Ovako Working Posture Analyzing System (OWAS) (Karhu et al., 1977), and to determine if these varied based on anthropometric and demographic characteristics. We hypothesized that the high-rise pack lift task would result in the greatest MSD risk because of the location and magnitude of the load as well as the tendency for firefighters to carry their pack over their shoulder.

## Methods

### Protocol Overview

A cohort of 48 firefighters were recruited from the Hamilton Professional Fire Fighters Association (HPFFA), a large urban city in Ontario, Canada, to perform three common hose-related firefighting tasks, (a) hose drag, (b) hose pull, and (c) high-rise pack lift and carry, at their local training center. Participants completed the tasks in full bunker gear, including a self-contained breathing apparatus (SCBA). Video observation was used to classify the postures during the initial hose pick-up (Tasks 1-3), hose manipulation (Tasks 1-3), and ambulation with the hose (Tasks 1 and 3).

### Participants

Active firefighters (*n* = 48) were recruited through email and volunteered to participate (note: unless explicitly discussing rank, the term “firefighters” will be used to refer to all participants in the present study.) All firefighters provided written, informed consent, which was approved by the Hamilton Integrated Research Ethics Board. Prior to performing the firefighting tasks, demographic (age, tenure, and rank) and anthropometric data (height and weight) were recorded. Participants were provided with a $15 gift card at the end of their participation.

### Experiment

Firefighters wore full bunker gear, which included their issued custom-sized jacket, pants, boots, gloves, and helmet (10.3 ± 0.80 kg), as well as a SCBA and mask (12.3 kg).

All tasks were performed in a standardized format, using set start positions and equipment. Participants were asked to start each task by standing within a 0.3 m × 0.4 m box marked by tape on the floor of the City of Hamilton Multi-Agency Training Academy. Depending on the task, either a hose nozzle (6.1 kg) attached to a single 15.24 m length of 38-mm fire hose (4.19 kg) or a high-rise pack (19.5 kg, containing two fire hose lengths, a hose nozzle, and a tool kit) were centered over-top of a marker to the right of the starting box, at a distance of 0.3 m from the side of the box and in-line with the front of the box.

**Figure 1.**
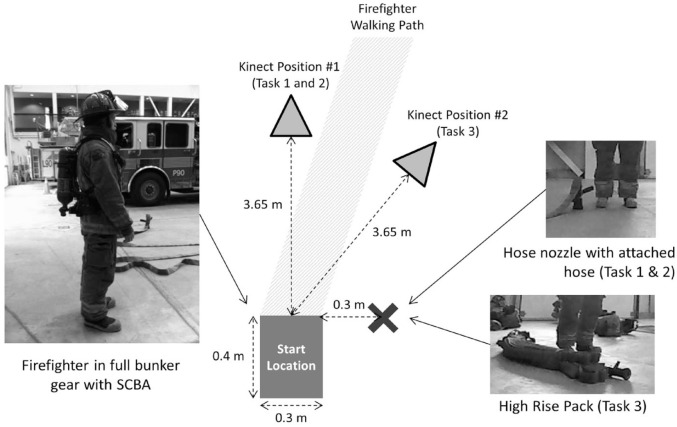
Diagram of experimental setup demonstrating positions of the equipment and participant locations (not to scale).

A Microsoft Kinect^®^ camera (Kinect for Xbox, Microsoft, WA, United States) was positioned at a height of 0.5 m and a distance of 3.65 m from the starting position in order to maximize the frontal plane view of the firefighters during task performance (Figure 1). Both 2D video data and 3D depth data were collected. For Tasks 1 and 2, the camera was located directly in front of the participants as they stood in their starting position. The camera was rotated by 35 degrees for Task 3 so that both the start posture and the high-rise pack pick-up posture could be adequately captured. Firefighters performed each task once in the same order as there was no risk of a learning effect given the regularity with which the tasks are performed in the same training environment.

### Tasks

The three tasks were selected based on discussions with HPFFA representatives who identified the tasks as common and challenging for firefighters. In addition, these tasks are part of the CPAT (Deakin et al., 1996) but were modified for the present study by reducing the space in which these movements were performed so they could be captured using video. Unlike with the CPAT exam, firefighters were asked to perform the tasks as they would at a standard fire call, rather than as quickly as possible in order to focus the investigation on posture assessment.

#### Task 1: Hose Drag Task

**Figure 2.**
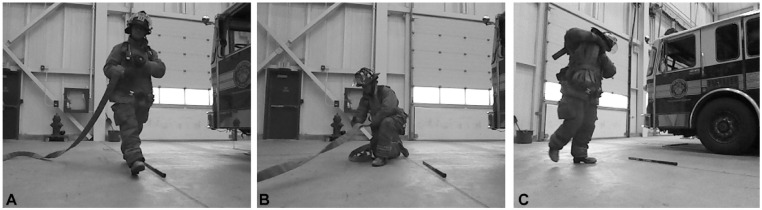
Sample images of the (A) hose drag (Task 1), (B) hose pull (Task 2), and (C) high-rise pack lift and carry (Task 3) tasks.

Firefighters were required to pick up a hose nozzle that was positioned standing on end at the standardized hose start location (Figure 2), and then take five steps forward while holding the hose nozzle. The hose was loosely coiled next to the nozzle. As per standard firefighting protocol, the hose was uncharged.

#### Task 2: Hose Pull Task

Firefighters were asked to pick up the nozzle and/or hose, which was positioned standing on end in the standardized hose start location, and draw the hose toward them using a hand-over-hand approach until five movements per hand had been performed. The hose was uncoiled in front of the start location. As per standard firefighting protocol, the hose was uncharged.

#### Task 3: High-Rise Pack Lift and Carry

The high-rise pack was centered over the standardized hose start location such that the length of the pack was parallel to the sagittal plane of the firefighter when in their starting position. Firefighters were asked to pick up the pack, place it on their shoulder, and take five steps in the forward direction.

### Video Data Collection and OWAS Analysis

Video data were collected using the Microsoft Kinect^®^ System (Kinect V1, Microsoft Corps., WA, United States). The Microsoft Kinect^®^ system collects both 2D video data using an RGB camera (640 × 480 pixel resolution) as well as 3D depth data using an infrared emitter and receiver (320 × 480 pixel resolution). The system is designed to capture data at a maximum of 30 Hz.

Video observation was used to classify the posture strategies at different phases of each task using the OWAS ergonomics tool (Karhu et al., 1977). OWAS is a full-body observational posture assessment tool with intra-rater reliability of 95% (Kee & Karwowski, 2007) and inter-rater reliability scores of 90% (Heinsalmi, 1986) and 93% (Karhu et al., 1977). A researcher with experience using video observation for ergonomics analysis performed the posture classification using the 2D video data from the Microsoft Kinect^®^ system. Where necessary, the 3D depth data were used to supplement the 2D data in order to most appropriately classify the posture strategies. The OWAS tool evaluates single static postures. Therefore, the video data were scanned and a single representative frame from each critical phase of each task was selected. The following phases for each of the three tasks were evaluated using the OWAS tool:

Task 1: hose initial contact (Phase 1), ambulation preparation (Phase 2), and ambulation with hose (Phase 3)
Task 2: hose initial contact (Phase 1) and hose pull (Phase 2)
Task 3: hose initial contact (Phase 1), hose lift to shoulder (Phase 2), and ambulation with hose (Phase 3)

The OWAS tool uses seven lower extremity posture categories, four trunk posture categories, three shoulder posture categories, and three load categories to determine where the overall observed postures fall within one of four action classification (AC) scores for postural intervention (1: no action needed, 2: action needed in the near future, 3: action needed as quickly as possible, and 4: immediate action required).

### Data Analysis

The SPSS software (IBM SPSS Statistics version 20, IBM, NY, United States) was used to conduct all statistical analyses. Descriptive statistics were calculated for all demographic (age, sex, rank, and tenure; response options in the appendix), anthropometric (height, weight, and BMI), and OWAS ergonomics evaluation tool components. The Wilcoxon signed-rank test was used to test for differences in OWAS AC scores between the task phases to determine which tasks required more urgent attention. The mode was determined to be the most common posture used to perform a task, which was used to determine which tasks require more attention. A Bonferroni correction was applied to alpha due to the 28 comparisons (α = .05/28 = .0018). This non-parametric test was selected given the ordinal and paired nature of the variables being compared. Chi-square analyses were used to determine if there were significant associations between each of the demographic and anthropometric variables and the OWAS AC scores. To reduce the frequency of having insufficiently populated analysis tables, the median value for each of the continuous variables was used to categorize the variables into two groups (i.e., low/high). The OWAS AC scores were divided into two groups: low priority (AC scores 1 and 2) and high priority (AC scores 3 and 4). Job rank reports were also divided into two groups: firefighters and captains (i.e., captains and acting captains). Significance was determined using the likelihood ratio Chi-square statistic given the small sample size with alpha corrected for the seven comparisons made for each task phase (α = .05/7 = .0071). The two-sided Fisher’s exact test was used when there was a failure to achieve 5 items per category in 20% of the categories.

## Results

Overall, 48 firefighters were recruited and completed this study (Table 1). Six of these firefighters were female. Seven male firefighters were Captains, and three male firefighters were Acting-Captains. The average age of the cohort was 43.0 years, with an average tenure of 14.8 years.

**Table 1.** Age, Job Tenure, and Physiological Variables by Gender (*N* = 48)

Variables		Male (*n* = 42)	Female (*n* = 6)	Full cohort (*N* = 48)
Age (years)	Mean (STD)	43.95 (8.82)	36.00 (5.44)	42.96 (8.84)
	Median	46.50	35.00	46.00
Tenure (years)	Mean (STD)	15.86 (8.70)	7.00 (3.62)	14.75 (8.73)
	Median	19.00	5.50	16.00
Height (cm)	Mean (STD)	179.78 (8.94)	167.68 (4.31)	178.27 (9.38)
	Median	180.34	168.91	179.07
Weight (kg)	Mean (STD)	96.51 (11.08)	69.99 (12.58)	93.20 (14.23)
	Median	95.16	65.45	95.16
BMI (kg/m^2^)	Mean (STD)	30.03 (4.33)	24.82 (3.83)	29.38 (4.57)
	Median	29.37	23.28	28.75
Rank (number of participants)	Firefighter	32	6	38
	Acting-captain	3	0	3
	Captain	7	0	7

*Note.* BMI = body mass index; STD = standard deviation.

The individual scoring components of the OWAS tool are shown in Supplemental Figure 1 to demonstrate the variability of observed postures. The overall mode (most common posture used to perform the tasks) of these OWAS results for each task phase is described in Table 2. A bent and twisted trunk posture was used by many firefighters in at least one of the task phases. Having a straight back was the most common posture for the ambulation phases (Phase 3) of Task 1 and Task 3. The two most commonly selected leg postures during Phase 1 for each task were standing or squatting on two bent legs and kneeling. Most firefighters chose to work with both arms below shoulder height, although having one or both arms at or above shoulder height was most common during Phases 2 and 3 of Task 3. Hand loads in this study were standardized because the same equipment was used for all firefighters. Thus, Tasks 1 and 2 required lifting a load under 10 kg, while Task 3 required lifting a load above 10 kg and equal to or less than 20 kg.

**Table 2.** OWAS Scores by Task and Task Phase

Positioning	Task 1 Hose drag			Task 2 Hose pull		Task 3 High-rise pack lift and carry		
	Phase 1	Phase 2	Phase 3	Phase 1	Phase 2	Phase 1	Phase 2	Phase 3
OWAS Item	Hose initial contact	Ambulation preparation	Ambulation with hose	Hose initial contact	Hose pull	Hose initial contact	Hose lift to shoulder	Ambulation with hose
Arms	Both arms below shoulder level	Both arms below shoulder level	Both arms below shoulder level	Both arms below shoulder level	Both arms below shoulder level	Both arms below shoulder level	Both arms at or above shoulder level	One arm at or above shoulder level
	100.0%	39.6%	70.8%	100.0%	100.0%	100.0%	97.9%	89.6%
Back	Bent and twisted	Straight	Straight	Bent and twisted	Bent and twisted	Bent	Bent and twisted	Straight
	54.2%	41.7%	97.9%	60.4%	68.8%	54.2%	64.6%	85.4%
Legs	Standing or squatting on two bent legs	Standing on two straight legs	Walking	Kneeling	Kneeling	Kneeling	Standing on two straight legs	Walking
	62.5%	62.5%	100.0%	50.0%	47.9%	54.2%	89.6%	100.0%
Load	≤10 kg	≤10 kg	≤10 kg	≤10 kg	≤10 kg	10 kg < x ≤ 20 kg	10 kg < x ≤ 20 kg	10 kg < x ≤ 20 kg
	100.0%	100.0%	100.0%	100.0%	100.0%	100.0%	100.0%	100.0%
AC score	Immediate action required	No action needed	No action needed	Immediate action required	Action needed in the near future	Immediate action required	Action needed as quickly as possible	No action needed
	45.8%	83.3%	97.9%	54.2%	39.6%	45.8%	56.3%	85.4%

*Note.* The mode was used to determine which postures and tasks are most performed by the firefighters and which postures pose a risk for injury. OWAS = Ovako Working Posture Analyzing System; AC = action classification.

The descriptive results from the OWAS assessment, as determined by the AC score, show that the initial contact phase of each task was the most problematic (Table 2). The most reported (i.e., mode) AC score for this phase for each task was 4 (45.8%, 54.2%, and 45.8% of the cases for Tasks 1, 2, and 3, respectively, Table 2), indicating that “immediate action” is needed to correct issues with the task in order to prevent MSDs. The high-rise pack lift-to-shoulder phase was identified as warranting action “as quickly as possible” (56.3% of cases), and the hose pull phase of Task 2 was identified as requiring action “in the near future” (39.6% of cases). The remainder of the phases identified that, in most cases, no action was required.

The Wilcoxon signed-rank test was used to identify the order of priority in addressing the ergonomics issues between task phases based on their OWAS AC scores (Table 3). The phases requiring the most urgent attention were the initial hose contact phases of each task. While no significant differences were found between each of these phases, Task 2 had a median AC score of 4, and Tasks 1 and 3 had median AC scores of 3 in the initial hose contact phase. Thus, Task 2 Phase 1 requires “immediate action,” whereas the other two task phases require action “as quickly as possible” but are not as urgent as Task 2 Phase 1. Task 3 Phase 2, or the phase where the high-rise pack was lifted to the shoulder, also requires action “as quickly as possible.” There was a statistical difference between Task 3 Phase 2 and Task 3 Phase 1 (*p* = .005). However, Task 3 Phase 2 ranks as a lesser priority compared with Phase 1 of the other two tasks (Task 1 Phase 1: *p* = .002; Task 2 Phase 1: *p* = .002). The only other task phase that needs to be addressed from an MSD prevention perspective (i.e., has an OWAS AC score above 1) is Task 2 Phase 2 (i.e., hose pull phase), which was identified as requiring action “in the near future.” Although this task is not statistically different from Task 3 Phase 2 (*p* = .580), it has a median OWAS AC score of 2 compared with an OWAS AC score of 3 for Task 3 Phase 2. Task 1 Phase 2 (ambulation preparation) and 3 (ambulation) and Task 3 Phase 2 (ambulation), each of which has an OWAS AC score of 1 indicating no action needed, are not different from each other but are significantly smaller than the other task phases.

**Table 3.** Wilcoxon Signed-Rank Test Results for Each Pair of Task Phase Comparisons of OWAS AC Scores

Tasks		Median OWAS action classification scores (top: row, bottom: column)							
		Task 1 Hose pull			Task 2 Hose drag		Task 3 High-rise pack lift		
		Phase 1	Phase 2	Phase 3	Phase 1	Phase 2	Phase 1	Phase 2	Phase 3
Task 1 Hose pull	Phase 1		13<0.001	13<0.001	430.95	23<0.001	330.92	33<0.001	13<0.001
	Phase 2			110.02	41<0.001	21<0.01	31<0.001	31<0.001	110.32
	Phase 3				41<0.001	21<0.001	31<0.001	31<0.001	110.01
Task 2 Hose drag	Phase 1					240.002	340.83	34<0.001	14<0.001
	Phase 2						32<0.001	320.58	12<0.001
Task 3 High-rise pack lift	Phase 1							330.005	13<0.001
	Phase 2								13<0.001
	Phase 3								
OWAS action priority rank (low [1]—high (4))		1	4	4	1	3	1	2	4

*Note.* The median OWAS scores for each pair of task phases are on the upper right of the table, with the *p* value of the comparison directly below these scores. For the median scores, the top value corresponds to the task phase in the respective column and the bottom value corresponds to the task phase in the respective row. Cells that are in bold demonstrate significant differences between compared task phase OWAS AC scores. Where significant differences lie, the associated task phase OWAS AC median scores provide insight into the ranked order of the pair. The bottom row contains the ranking of ergonomics action prioritization (highest to lowest) based on the 28 comparisons of the Wilcoxon signed-rank test. OWAS = Ovako Working Posture Analyzing System; AC = action classification.

Associations between OWAS AC scores and independent variables, including demographic and anthropometric variables, were examined using a chi-square analysis (Supplemental Table 1). The only significant association was BMI with the Task 3 Phase 1 OWAS AC score, where a BMI of less than 28.75 was associated with a high AC score (likelihood ratio: 9.20, *p* = .002). No other significant associations were identified. The Chi-square analysis could not be run for Task 1 Phase 3 because all the OWAS AC scores fell into the “low AC score” creating an incomplete matrix for the analysis.

## Discussion

The evaluation of three common simulated fire suppression tasks has identified that hose manipulation at ground level or lifting to shoulder height, rather than simple movement or ambulation with the hose, requires ergonomics attention to reduce or prevent MSDs to firefighters. Overall, Task 3 was identified as most urgently needing ergonomics action. These findings are congruent with recent research by Frost et al. (2016) where bending, lifting, and/or squatting were the movements most frequently associated with firefighter MSDs (Frost et al., 2016). Indeed, in order to lift a hose up off the ground, as is needed in each task assessed herein, either bending or squatting combined with a lifting movement is required. Gentzler and Stader (2010) used observational methods and ergonomics tools (NIOSH lifting equation, RULA, and REBA) to study post-fire tasks (hose lift to drain and hose rolling) (Gentzler & Stader, 2010). Their posture-related analysis highlighted similar trends to those found in the current study, where lifting above the shoulder and working with hoses at ground level were reported as being high to very high MSD risk levels.

A closer examination of the OWAS tool sub-components suggests that a better understanding of posture mechanics during each task phase is needed. Common concerns across phases included bent and twisted back postures, and kneeling, squatting, and standing with two bent legs. The contribution of these back and leg postures to the high OWAS AC scores is supported by the general movement patterns used by Calgary firefighters at the time of injury (Frost et al., 2016). In Task 3, additional factors contributing to the need for ergonomics action included working with one or both arms at or above shoulder height and the greater load of the high-rise pack. Interestingly, Frost et al. (2016) reported that shoulder MSDs in Calgary firefighters occurred during non-fire-related activities, such as training at the firefighter training center, performing physical training, fulfilling fire station duties, or attending to non-fire-related emergency calls (Frost et al., 2016). Since the tasks evaluated in this study were simulated fire suppression tasks performed within the context of the training center, we can infer, based on the report by Frost et al. (2016), that the observed postures were performed with no less MSD potential than they would at a real fire (Frost et al., 2016). Therefore, the selected tasks are good examples of the postures that may lead to MSDs in this workforce. However, to be certain of this inference, a similar evaluation of the MSD statistics conducted at the Calgary Fire Department is needed for the HPFFA.

### Associations Between Ergonomics Evaluations and Firefighter Characteristics

Results from this study indicated that firefighter anthropometric and demographic characteristics were not associated with posture selection during the three simulated fire suppression tasks. The exception was the association between BMI and the final OWAS AC score for Task 3 Phase 1, where a low BMI was associated with a high OWAS AC score or a more urgent need for ergonomics action. This association was opposite to previous studies (Jahnke et al., 2013; Neitzel et al., 2016). Given that neither height nor weight was associated with the final OWAS AC score, increased muscle mass, and therefore strength, rather than excess body fat, may explain the current association between higher BMI and choosing less risky postures. Alternatively, the individual may have more lean body mass suggesting familiarity with resistance training and improved technique while lifting. Indeed, a lower percentage of body fat has been associated with greater firefighter fitness (Poplin et al., 2016) and better performance on the Firefighter’s Ability Test (Michaelides et al., 2008).

Age and the associated variable of tenure were hypothesized to be associated with final OWAS AC scores, although the current results suggested the direction of this relationship was uncertain. We considered that increased age might translate into more opportunity to learn or hone in on safer postures, which would lead to lower OWAS AC scores. Alternatively, we also expected that more recent health and safety training as a recruit might improve rates of safe posture selection in junior firefighters. Although no association between firefighter age and MSD risk was found in the current study, there is conflicting evidence in the literature about the relationship between these two variables, particularly with respect to low back pain (Katsavouni et al., 2014; Lett & McGill, 2006). However, an analysis of an estimated 66,200 injuries reported to the National Fire Incident Reporting System shows that most injuries occur in firefighters between 40 and 44 years of age, with fewer injuries occurring in younger and older workers (U.S. Department of Homeland Security, 2011). Thus, a more sophisticated analysis is needed to determine how age is associated with firefighter MSD risk.

Sex and job category were also expected to be associated with OWAS AC scores, but this relationship was not found in the current study. Past studies, however, demonstrate that female firefighters are at a greater risk of low back pain than their male counterparts, and firefighter officers have a decreased risk of low back pain compared with fire truck drivers (Negm et al., 2017). Thus, the risk is likely associated with the tasks not the job category or firefighter characteristics per se. In drawing conclusions from our study, it is important to note that, while the posture-specific OWAS AC scores are used as a surrogate for MSD risk, there are other risk factors to consider. These factors may be task-related, such as handle height for pushing and pulling (Lett & McGill, 2006), fitness-related, such as flexibility (Neitzel et al., 2016; Poplin et al., 2016), or psychosocial such as perceived job demands (Neitzel et al., 2016).

### Study Limitations

Overall, the OWAS tool is aligned with firefighter ergonomics assessment needs because it focuses on commonly injured body regions including the back, arms, and legs (Frost et al., 2016; Kajaks & & MacDermid, 2015; U.S. Department of Homeland Security, 2011; WSIB, 2016). Nevertheless, there are limitations to using OWAS that need to be acknowledged. Specifically, the OWAS tool uses (a) minimal and general posture categories with broad bin definitions and (b) limited load exposure factors to evaluate the need for ergonomics action (David, 2005). However, given the high intra- and inter-rater reliability scores of over 90% (Heinsalmi, 1986; Karhu et al., 1981; Kee & Karwowski, 2007), the broad bin boundaries suggest misclassification of postures is less of a concern with the OWAS tool compared with other posture-bin tools (Andrews et al., 2008). Furthermore, the unpredictable work demands of firefighters make it difficult to reliably determine or estimate load exposure variables over the course of a work shift without additional data collection using more sophisticated equipment. As well, we asked the firefighters to perform the tasks as they would at a standard fire call, rather than as quickly as possible to focus the investigation on posture assessment. Thus, the OWAS tool is valuable for an initial ergonomics assessment of simulated firefighter tasks to determine where more advanced investigations are needed.

Additional methodological limitations include using a single representative static posture for task phase evaluation, and not accounting for the body-borne external loads. A more dynamic assessment of firefighters wearing complete bunker gear during manual materials handling tasks would have allowed for the consideration of potentially important inertial properties of the system. However, while this is a limitation of the OWAS tool, current ergonomics tools are not yet advanced enough to conduct this analysis (Yunus et al., 2021). Furthermore, the degree to which the single trial evaluated is representative of the actual postures used by the firefighters in both training and real fire suppression tasks is questionable. We made an *a priori* assumption that firefighter experience and task simplicity warranted asking firefighters to perform each task one time. Future work should evaluate the reproducibility of the observed firefighter postures in real training or active fire suppression environments.

Finally, a sample of 48 firefighters (*n* = 6 female) volunteered to participate from a cohort of 471 total full-time firefighters (*n* = 13 female). This sample was small for the more sophisticated statistical analyses that may have otherwise allowed for associations between firefighter characteristics and OWAS AC scores to be determined.

### Implications for Occupational Health Practice

The over-arching motivation for conducting this study was to inform the development of firefighter ergonomics training programs. Previous research related to firefighter MSD prevention has largely focused on identifying demographic, anthropometric, and health-related risk factors (Jahnke et al., 2013; Michaelides et al., 2008; Neitzel et al., 2016; Poplin et al., 2016). Few studies, however, have used the more traditional ergonomics assessment approach and focused on firefighter movement and posture mechanics. Those who have investigated the relationship between firefighter MSD risk and movement mechanics have done so using tasks that are not directly work-related, such as generic pushing and pulling tasks (Lett & McGill, 2006) or fitness training programs (Beach et al., 2014; Frost et al., 2015). The relevance of these study findings to MSD prevention during real or training-related fire suppression tasks has not been clearly demonstrated. In fact, Beach et al. (2014) found that, while fitness measures improved as a result of a 12-week exercise program, these improvements were not observed in the Functional Movement Screen^TM^ scores or occupational low back loading measures (Beach et al., 2014). As a result, a purely fitness-based intervention for MSD prevention in firefighters was not recommended. However, Frost et al. (2015) did find improvements in outcomes that are more transferable to the work-related activities performed at fires (i.e., fireground activities), such as spine and knee motion control, in firefighters undergoing a 12-week movement-guided fitness program rather than a conventional fitness program (Frost et al., 2015). The approach shows promise as a behavioral learning method that engrains safer movement patterns into the regular duties of firefighters.

Thus, a movement-oriented approach to the training of high-MSD risk tasks grounded in contextually relevant body mechanics education, with resistance training that supports task-specific movements may teach behaviors that will ultimately lead to safer movement strategy during firefighting. The first step in developing such a training program is to better understand which, and why, tasks place firefighters at a high risk of MSDs. The current study provides detailed insights into three common fire suppression tasks. Future research will focus on developing a training program that incorporates study findings. For those tasks requiring ergonomics action, in-depth investigations using more sophisticated ergonomics tools and kinematic analyses (Sinden et al., 2016) is needed to determine how the tasks can be improved. Specifically, we intend to use the 3D data collected with the Microsoft Kinect System® to drive the movements of digital human models embedded in advanced ergonomics assessment software. Further research is also needed to better understand if and how postures differ between training environments and those used during emergency and non-emergency calls. This will help us determine if personalized training programs based on firefighter characteristics (e.g., anthropometrics and demographics) are needed. Finally, in addition to focusing on better training for firefighters, there should be parallel efforts looking at ways to re-design the tasks and equipment used to make the work environment as safe as possible for firefighters given their high risk of MSDs.

## Conclusion

Firefighters require high physical, cardiovascular, and cognitive demands in their jobs. Unfortunately, this job can leave firefighters vulnerable to MSDs, among other health complications. Improved ergonomics training programs may help reduce these MSDs. This work provides insight into which phases of three common fire suppression tasks may place firefighters at risk of MSDs, which is an important first step in gathering the information needed to develop more effective injury prevention ergonomics training programs for firefighters.

**Applying Research to Occupational Health Practice**

The over-arching motivation for conducting this study was to inform the development of firefighter ergonomics training programs. A movement-oriented approach to the training of high-MSD risk tasks grounded in contextually relevant body mechanics education, with resistance training that supports task-specific movements may teach behaviors that will ultimately lead to safer movement strategy during firefighting. This work provides insight into which phases of three common fire suppression tasks may place firefighters at risk of MSDs, which is an important first step in gathering the information needed to develop more effective injury prevention ergonomics training programs for firefighters.

## Supplemental Material

sj-png-1-whs-10.1177_21650799231214275 – Supplemental material for Posture Evaluation of Firefighters During Simulated Fire Suppression Tasks Click here for additional data file. Supplemental material, sj-png-1-whs-10.1177_21650799231214275 for Posture Evaluation of Firefighters During Simulated Fire Suppression Tasks by Tara Kajaks, Christina Ziebart, Vickie Galea, Brenda Vrkljan and Joy C. MacDermid in Workplace Health & Safety
