# Supplementary figures and images for: Posture Evaluation of Firefighters During Simulated Fire Suppression Tasks
Source: Workplace Health Saf. 2023 Nov 24;71(12):606–16. doi: 10.1177/21650799231214275 (PMC10676042; doi:10.1177/21650799231214275)

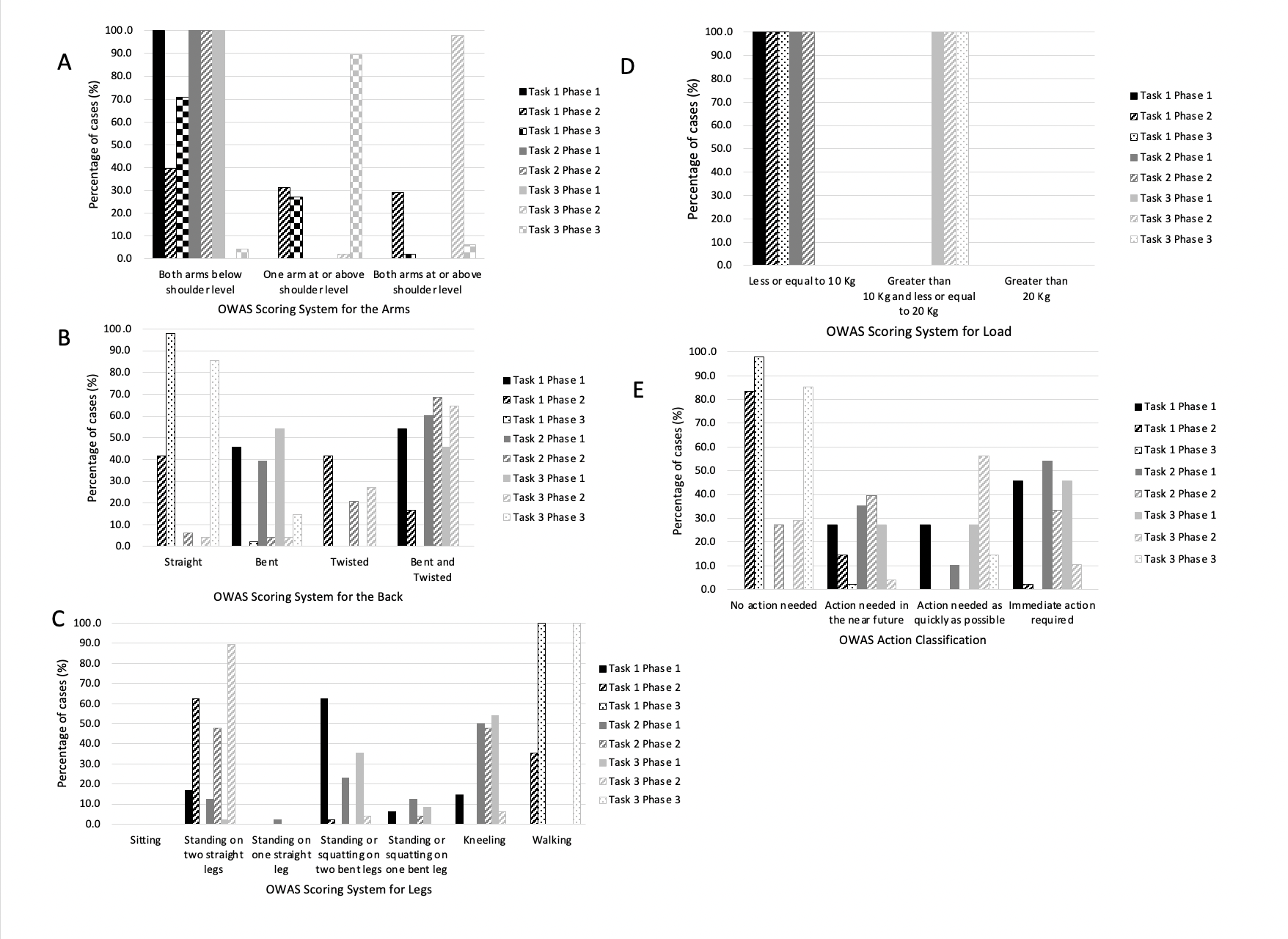

Supplement: sj-png-1-whs-10.1177_21650799231214275 – Supplemental material for Posture Evaluation of Firefighters During Simulated Fire Suppression Tasks [file sj-png-1-whs-10.1177_21650799231214275.png]
